# Physical Activity of Poles in the Care for Their Health Potential Before and During the COVID-19 Pandemic

**DOI:** 10.1017/dmp.2020.398

**Published:** 2020-10-22

**Authors:** Kamil Zaworski, Zofia Kubińska, Adrianna Dziewulska, Olga Walasek

**Affiliations:** Pope John Paul II State School of Higher Education in Biała Podlaska, Department of Physiotherapy, Biała Podlaska, Poland

**Keywords:** exercise, health behavior, pandemics, physical activity

## Abstract

**Objectives:**

The aim of this study is to present the engagement of adult Poles in physical activity (PA) before and during the coronavirus pandemic, taking into consideration: frequency, duration, and types of the activity, depending on the gender and age of the participants.

**Methods:**

The study was conducted using an online survey questionnaire. A total of 688 residents of Poland aged 18 to 58 (28.61 ± 9.5) y participated in the study.

**Results:**

A statistically significant decrease in the frequency of PA was noted in the group of men (*P* = 0.0001) and in the age group of 39 to 58 y old. The analysis of the duration of a single PA before and during the pandemic has shown a statistically significant reduction in the workout time among both men and women and across all age groups (*P* = 0.05). There was a statistically significant increase in the frequency of women undertaking flexibility exercises, eg, yoga (*P* = 0.000), as well as a decrease in marching and walks (*P* = 0.003). Men significantly less frequently did strength exercises (*P* = 0.002).

**Conclusions:**

During the pandemic, there was a statistically significant decrease in the frequency and duration of PA. The preferences of the participants as to the type of PA undertaken changed as well.

Since early 2020, the world has been struggling with the coronavirus disease 2019 (COVID-19) pandemic caused by the severe acute respiratory syndrome coronavirus 2 (SARS-CoV-2) coronavirus. On March 15, 2020, the Polish government introduced a ban on movement of people within the country, which likely had a significant impact on the reduction of daily physical activity (PA) of the entire population. On April 8, the Chief Sanitary Inspectorate advised Poles to refrain from any outdoor PA during the epidemic.

Therefore, according to Hall et al., this is a situation of 2 pandemics occurring simultaneously. Although the end of the COVID-19 pandemic will enable a return to the so-called normal life, the pandemic of physical inactivity will continue, and as a result of COVID-19, we may face an aggravation of the problem of physical inactivity among the population.^1^

Regular engagement in PA reflects the acquired health competences: knowledge, skills, beliefs, attitudes and recognized needs with regard to taking care of one’s health.^2^

The pandemic forced changes in the functioning of many sectors of economy and education, such as remote work and learning, which additionally generate an increase in sedentary behaviors. Due to the limited opportunities to engage in PA outdoors, a vast majority of the participants stayed at home to prevent the spread of the virus by means of self-isolation and quarantine. The introduction of regular physical exercise in home environment during the pandemic would significantly reduce the risks to physical and mental health.^3^

The aim of this study is to present the engagement of adult Poles in PA before and during the coronavirus pandemic, taking into consideration: frequency, duration, types, and health benefits (of PA), depending on the gender and age of the participants.

## Methods

The study was conducted from April 14 to 30, 2020, using a survey questionnaire posted in social media. The original survey questionnaire consisted of 4 metric questions (age, gender, place of residence, education), and questions on selected aspects of respondents’ participation in PA before and during the COVID-19 pandemic, such as frequency, duration, types, and expected benefits (the questionnaire is available in Supplemental Materials, which are available online). Polish citizens between 18 and 65 y of age who had access to the Internet/Facebook and wanted to participate in the anonymous survey for scientific purposes on their own initiative could participate in the survey. Before the survey, each participant was acquainted with the principles of data collection and protection and gave informed consent to participation.

A total of 688 residents of Poland aged 18 to 58 y (average age, 28.61 ± 9.5 y) participated in the study, including 491 (71.0%) women and 197 (29.0%) men. A total of 473 (69.0%) of them live in a urban areas and 215 (31.0%) in rural areas. The majority of the respondents (373 [55%] persons) had higher education, 288 (42%) secondary education, 12 (2%) basic education, and 1 (1%) vocational education.

Qualitative variables are described using the number of observations with the variant of a specific characteristic (n) and the corresponding percentage. The Lilliefors test and the Shapiro-Wilk test were used to verify the normality of variable distribution. The relationship between the variables before and during the pandemic was measured using the Wilcoxon signed-rank test. The correlation between the variables on an ordinal scale was assessed using Spearman’s rank correlation coefficient (Spearman’s ρ). The correlation between the variables on a nominal scale was measured using the chi-squared test with Fisher’s correction and Pearson’s C and Cramer’s V coefficients.

The analysis was conducted using the IBM SPSS 25.0 software. Interdependencies/correlations/differences were considered statistically significant if *P* ≤ 0.05.

## Results

The analysis of the frequency of engaging in PA before and during the pandemic has shown a statistically significant decrease in the frequency of PA in the group of men (*P* = 0.0003) and in the age group of 39 to 58 y (*P* = 0,001). Men indicated less frequently that during the pandemic they practiced PA daily (19.3% to 10.2%), while the number of the oldest people not practicing any PA before the pandemic increased from 1.7% to 19.5%. There was no statistically significant decrease in the frequency of physical activity in the group of women (*P* = 0.479) and persons aged 18-28 y (*P* = 0.75) and 29-38 y (*P* = 0.676). Detailed results are presented in Supplemental Table 1. In addition, a correlation between the frequency of engaging in PA and the participants’ age during the pandemic (r = −0.09; *P* = 0.0004) was found: the younger the participants, the more often they engaged in PA.

The analysis of the duration of a single PA before and during the pandemic has shown a statistically significant shortening of the workout time among both men and women as well as in all age groups (*P* < 0.05) (Table 1). Also of statistical significance was the correlation between age and duration of a single PA before (r = −0.124; *P* = 0.001) and during the pandemic (r = −0.116; *P* = 0.003); the younger the individual, the longer the PA.

**Table 1.** Duration of a single PA undertaken by the participants before and during the pandemic (*n* = 688)

Sex						
Variable	Variants	Before Pandemic		During Pandemic		*P*-Value
		*n*	%	*n*	%	
Women (*n* = 491)	None	28	5.7	43	8.8	0.0004*
	Up to 10 min	12	2.4	22	4.5	
	10 to 15 min	23	4.7	33	6.7	
	15 to 30 min	94	19.1	94	19.1	
	30 to 60 min	171	34.8	205	41.8	
	60 to 90 min	111	22.6	73	14.9	
	Above 90 min	42	8.6	21	4.3	
Men (*n* = 197)	None	9	14.6	22	11.2	0.0002*
	Up to 10 min	4	2	8	4.1	
	10 to 15 min	4	2	11	5.6	
	15 to 30 min	14	7.1	33	16.8	
	30 to 60 min	44	22.3	54	27.4	
	60 to 90 min	69	35	45	22.8	
	Above 90 min	53	26.9	24	12.2	
Age Group						
18–28 y (*n* = 435)	None	27	6.2	30	6.9	0.0004*
	Up to 10 min	10	2.3	14	3.2	
	10 to 15 min	16	3.7	26	6	
	15 to 30 min	65	14.9	88	20.2	
	30 to 60 min	117	26.9	160	36.8	
	60 to 90 min	120	27.6	85	19.5	
	Above 90 min	80	18.4	32	7.4	
29–38 y (*n* = 135)	None	9	6.7	16	11.9	0.049*
	Up to 10 min	3	2.2	8	5.9	
	10 to 15 min	5	3.7	6	4.4	
	15 to 30 min	24	17.8	19	14.1	
	30 to 60 min	50	37	58	43	
	60 to 90 min	36	26.7	20	14.8	
	Above 90 min	8	5.9	8	5.9	
39-58 y (*n* = 118)	None	3	2.5	23	19.5	0.0003*
	Up to 10 min	4	3.4	7	5.9	
	10 to 15 min	9	7.6	10	8.5	
	15 to 30 min	19	6.1	20	16.9	
	30 to 60 min	48	40.7	42	35.6	
	60 to 90 min	26	22	13	11	
	Above 90 min	9	7.6	3	2.5	

* Statistically significant data.

The participants engaged in various types of PA. There was a statistically significant increase in the frequency of women undertaking flexibility exercises, eg, yoga (*P* = 0.0004), as well as a decrease in marching and walks (*P* = 0.003). Men performed strength exercises significantly less frequently (*P* = 0.002). As far as the age of the participants is concerned, flexibility exercises were performed significantly more frequently by the youngest group (*P* = 0.0002). Participants from the youngest group (*P* = 0.04) and the middle-aged group (*P* = 0.03) reported a significantly lower frequency of walks, whereas participants from the oldest group did so for strength exercises (*P* = 0.002) (Table 2).

**Table 2.** Types of PA undertaken before and during the pandemic (*n* = 688)

Sex						
Variable	Variants	Before Pandemic		During Pandemic		*P*-Value
		*n*	%	*n*	%	
Women (*n* = 491)	Jogging	89	18.1	86	17.5	0.854
	Flexibility exercises/yoga	81	16.5	122	24.8	0.0004*
	Strength exercises	163	33.2	140	28.5	0.092
	Fitness/Pilates/aerobic	172	35	165	33.6	0.654
	General improvement exercises	107	21.8	126	25.7	0.111
	Marching, walks	284	57.8	236	48.1	0.003*
	Nordic walking	17	3.5	12	2.4	0.322
	Cycling/roller skating	133	27.1	123	25.1	0.452
	None	28	5.7	47	9.6	0.019*
Men (*n* = 197)	Jogging	62	31.5	45	22.8	0.078
	Flexibility exercises/yoga	13	6.6	19	9.6	0.276
	Strength exercises	122	61.9	93	47.2	0.002*
	Fitness/Pilates/aerobic	13	6.6	9	4.6	0.497
	General improvement exercises	32	16.2	41	20.8	0.138
	Marching, walks	81	41.4	71	36	0.277
	Nordic walking	0	0	0	0	-
	Cycling/roller skating	57	28.9	47	23.9	0.264
	None	10	5.1	26	13.2	0.001*
Age Group						
18–28 y (*n* = 435)	Jogging	105	24.1	93	21.4	0.329
	Flexibility exercises/yoga	62	14.3	113	26	0.0002*
	Strength exercises	215	49.4	179	41.4	0.005*
	Fitness/Pilates/aerobic	125	28.7	122	28	0.728
	General improvement exercises	90	20.7	107	24.6	0.118
	Marching, walks	233	53.6	203	46.7	0.036*
	Nordic walking	6	1.4	6	1.4	0.841
	Cycling/roller skating	124	28.5	114	26.2	0.423
	None	24	5.5	36	8.2	0.108
29–38 y (*n* = 135)	Jogging	30	22.2	27	20	0.746
	Flexibility exercises/yoga	18	13.3	16	11.9	0.669
	Strength exercises	36	26.7	38	28.1	0.627
	Fitness/Pilates/aerobic	36	26.7	36	26.7	0.878
	General improvement exercises	27	20	33	24.4	0.332
	Marching, walks	71	52.6	56	41.5	0.026*
	Nordic walking	5	3.7	5	3.7	0.637
	Cycling/roller skating	33	24.4	31	23	0.754
	None	10	7.4	16	11.9	0.094
39-58 y (*n* = 118)	Jogging	16	13.6	11	9.3	0.641
	Flexibility exercises/yoga	14	11.9	12	10.2	0.625
	Strength exercises	34	28.8	16	13.6	0.002*
	Fitness/Pilates/aerobic	24	20.3	16	13.6	0.276
	General improvement exercises	22	18.6	27	22.9	0.289
	Marching, walks	61	51.7	48	40.7	0.189
	Nordic walking	6	5.1	1	0.8	0.072
	Cycling/roller skating	33	28	25	21.2	0.315
	None	4	3.4	21	17.8	0.0004*

* Statistically significant data.

An analysis of the expected beneficial health effects resulting from engaging in PA during the pandemic has shown that both women and men indicated improved mental health, among others, significantly more frequently and staying fit and maintaining a good figure less frequently (*P* < 0.05). All the sample groups, in terms of gender and age, were more likely to expect that PA during the pandemic would contribute to alleviate lockdown-related isolation fatigue (*P* < 0.01) (Supplemental Table 2).

## Discussion

Government-imposed and systemic restrictions (lockdown, quarantine) introduced to inhibit the spread of SARS-CoV-2 infections caused a decrease in the level of PA undertaken by the population due to temporary ban on movement and on the use of public outdoor areas and PA facilities (group exercise classes, swimming pools, gyms).^4^ According to our studies, during the pandemic, there was a statistically significant decrease in the frequency of engaging in PA in the group of men and in the oldest age group. Although the COVID-19–related restrictions are aimed to protect the health of the elderly, social distancing and isolation may have a negative impact on their physical and mental health, as confirmed by other authors.^5^

A study by Qin et al. conducted on a group of 12,107 Chinese residents showed that 60.0% of the participants had an insufficient level of PA. The authors also highlight a lower level of PA among women and the largest decrease in PA among young people aged 20 to 34 y.^6^ In our own research during the pandemic, the duration of PA in the group of women and young people was significantly shortened, but the frequency of the sessions did not change substantially.

Our studies have shown a significant decrease of engagement in marching and walks in the group of women and in the group of young and middle-aged individuals, probably as a result of the government-imposed restrictions. These findings are confirmed by Ammar et al.,^4^ who observed a 34.0% decrease in walks during quarantine in a study conducted on 1047 surveyed participants.

It is worth noting that, according to our study, improving physical fitness and figure during the pandemic are not expected as frequently as other benefits by either gender group. This may be related to the increase of Poles’ body weight and body mass index (BMI) values. The BMI is an important indicator of lung capacity, respiratory mechanics, and oxygenation. This is of importance not only in everyday life, but especially during COVID-19 treatment with mechanical ventilation. Therefore, patients with obesity or diabetes can be particularly prone to respiratory failure and complications during such medical interventions.^7^ Preventive physiotherapy with PA can improve respiratory parameters in patients and increase their chances of survival.

During mandatory isolation, due to the risk of a decrease in the PA level, it is necessary to introduce at-home workout routines, adequate to the surroundings and abilities, planned by medical specialists, eg, physiotherapists. The recommended training routine should include strength, flexibility, aerobic and balance exercises, as well as walking.^5^

The results of the own research indicate that flexibility exercises are performed more often during a pandemic by women and young people compared with the period before (or prior) the pandemic. As mentioned earlier, strength exercises were also undertaken significantly less frequently by men and young and elderly people compared with the prepandemic period.

In their publication, Halabchi et al.^8^ and Laddu et al.,^9^ citing studies of other researchers, indicate that physical effort of moderate intensity has a positive effect on the immune system in a healthy individual in the context of viral infections. However, a highly intensive training routine weakens the functions of the immune system for several hours after the effort ends. These conclusions apply to active, healthy individuals with good immunity and not to the entire population. Implementation of PA in individuals with COVID-19 symptoms requires carefulness, close observation, and tests.^8^ Of interest, in their own research,^8^ only 1 in 5 respondents indicated among the expected effects of PA, strengthening the body’s immunity, which may be a consequence of lack of knowledge on this subject.

The promotion of preventive physiotherapy by PA is a priority factor for maintaining good health in individual and public terms. Knowledge on the subject, provided to the public through social campaigns involving a doctor and a physiotherapist, is a powerful, trust-based motivator.

As long as we do not have an effective cure and vaccine for SARS-CoV-2, experts recommend promoting: rational nutrition, hygienic lifestyle, and, above all, regular PA. We have a real influence on how these factors enhancing human physical and mental health are implemented.^4^ The most important aspect of PA in terms of health improvement during the pandemic, as confirmed by experts, is its regularity.^10^

Due to the tool used (original survey questionnaire), our own research is characterized by many limitations. The eligibility criteria include only basic data. For example, the level of patients’ awareness of the health benefits of PA and other reasons for reducing PA levels, such as taking care of another person, were not considered.

## Conclusions

In the surveyed group of Poles, there was a statistically significant decrease in the frequency of practicing PA during the pandemic in the group of men and the oldest people, and a reduction in the time of 1-time PA occurred in all analyzed groups. Most of the respondents indicated the improvement of mental health as the benefit of PA during the pandemic, and less frequently the improvement of the figure and condition, ie, physical health. Due to its numerous health benefits, PA is recommended to be promoted in all social groups affected by the SARS-CoV-2 pandemic.

## Supplementary material

For supplementary material accompanying this paper visit https://doi.org/10.1017/dmp.2020.398. click here to view supplementary material
