# Supplementary material for: Physical Activity of Poles in the Care for Their Health Potential Before and During the COVID-19 Pandemic
Source: Disaster Med Public Health Prep. 2020 Oct 22:1–4. doi: 10.1017/dmp.2020.398 (PMC7889839; doi:10.1017/dmp.2020.398)
Supplement: Supplementary file 1 [file S1935789320003985sup001.docx]

**Table 1. Frequency of PA before and during the pandemic according to the sex and age of participants (n=688)**

| **Sex** | | | | | | |
| --- | --- | --- | --- | --- | --- | --- |
| **Variable** | Variants | Before pandemic | | During pandemic | | p |
|  |  | n | % | n | % |  |
| **Women (n=491)** | Daily | 56 | 11.4% | 116 | 23.6% | 0.479 |
|  | Several times a week | 235 | 47.9% | 183 | 37.3% |  |
|  | Once a week | 70 | 14.3% | 63 | 12.8% |  |
|  | Several times a month | 59 | 12% | 57 | 11.6% |  |
|  | Less than once a month | 35 | 7.1% | 27 | 5.5% |  |
|  | None | 36 | 7.3% | 45 | 9.2% |  |
| **Men (n=197)** | Daily | 38 | 19.3% | 20 | 10.2% | 0.0003* |
|  | Several times a week | 104 | 52.8% | 88 | 44.7% |  |
|  | Once a week | 19 | 9.6% | 35 | 17.8 |  |
|  | Several times a month | 18 | 9.1% | 18 | 9.1% |  |
|  | Less than once a month | 11 | 5.6% | 16 | 8.1% |  |
|  | None | 7 | 3.6% | 20 | 10.2% |  |
| **Age group** | | | | | | |
| **18–28 (n=435)** | Daily | 59 | 13.6% | 90 | 20.7% | 0.750 |
|  | Several times a week | 231 | 53.1% | 175 | 40.2% |  |
|  | Once a week | 46 | 10.6% | 67 | 22.3% |  |
|  | Several times a month | 52 | 12% | 52 | 12% |  |
|  | Less than once a month | 22 | 5.1% | 20 | 4.6% |  |
|  | None | 25 | 5.7% | 31 | 7.1% |  |
| **29–38 (n=135)** | Daily | 19 | 14.1% | 27 | 20% | 0.676 |
|  | Several times a week | 63 | 46.7% | 59 | 43.7% |  |
|  | Once a week | 18 | 13.3% | 16 | 11.9% |  |
|  | Several times a month | 16 | 11.9% | 12 | 8.9% |  |
|  | Less than once a month | 9 | 6.7% | 8 | 5.9% |  |
|  | None | 10 | 7.4% | 13 | 9.6% |  |
| **39-58 (n=118)** | Daily | 21 | 17.8% | 18 | 15.3% | 0.001* |
|  | Several times a week | 51 | 43.2% | 36 | 30.5% |  |
|  | Once a week | 22 | 18.6% | 16 | 13.6% |  |
|  | Several times a month | 9 | 7.6% | 13 | 11% |  |
|  | Less than once a month | 13 | 11% | 12 | 10.2% |  |
|  | None | 2 | 1.7% | 23 | 19.5% |  |

*statistically significant data

**Table 2. Expected benefits resulting from engaging in PA (n=688)**

| **Sex** | | | | | | |
| --- | --- | --- | --- | --- | --- | --- |
| **Variable** | Variants | Before pandemic | | During pandemic | | p |
|  |  | n | % | n | % |  |
| **Women (n=491)** | No specific benefits in mind | 57 | 11.6% | 46 | 9.4% | 0.224 |
|  | Improved immunity | 125 | 25.5% | 126 | 25.7% | 0.704 |
|  | Improved general well-being | 316 | 64.3% | 295 | 60% | 0.261 |
|  | Improved mental health | 184 | 37.5% | 242 | 49.3% | 0.0003* |
|  | Escape from daily routine | 68 | 13.8 | 171 | 34.8% | 0.0003* |
|  | Staying fit, in shape | 334 | 68% | 298 | 60.7% | 0.019* |
|  | Pain alleviation | 76 | 15.5% | 75 | 15.3% | 0.890 |
|  | Stress reduction | 167 | 34% | 157 | 32% | 0.453 |
|  | Reduced lockdown fatigue | 19 | 3.9% | 179 | 36.5% | 0.0001* |
| **Men (n=197)** | No specific benefits in mind | 20 | 10.2% | 24 | 12.2% | 0.462 |
|  | Improved immunity | 43 | 21.8% | 43 | 21.8% | 0.879 |
|  | Improved general well-being | 124 | 62.9% | 111 | 56.3% | 0.193 |
|  | Improved mental health | 73 | 37.1% | 85 | 43.1% | 0.029* |
|  | Escape from daily routine | 61 | 31% | 67 | 34% | 0.466 |
|  | Staying fit, in shape | 144 | 73.1% | 123 | 62.4% | 0.005* |
|  | Pain alleviation | 14 | 7.1% | 16 | 8.1% | 0.668 |
|  | Stress reduction | 82 | 41.6% | 62 | 31.5% | 0.016* |
|  | Reduced lockdown fatigue | 9 | 4.6% | 65 | 33% | 0.0004* |
| **Age group** | | | | | | |
| **18–28 (n=435)** | No specific benefits in mind | 54 | 12.5% | 41 | 9.6% | 0.136 |
|  | Improved immunity | 96 | 22.3% | 109 | 25.4% | 0.206 |
|  | Improved general well-being | 270 | 62.6% | 264 | 61.5% | 0.820 |
|  | Improved mental health | 166 | 38.5% | 219 | 51% | 0.0002* |
|  | Escape from daily routine | 102 | 23.7% | 183 | 42.7% | 0.0004* |
|  | Staying fit, in shape | 314 | 72.9% | 290 | 67.6% | 0.061 |
|  | Pain alleviation | 56 | 13% | 60 | 14% | 0.598 |
|  | Stress reduction | 169 | 39.2% | 143 | 33.3% | 0.0002* |
|  | Reduced lockdown fatigue | 19 | 4.4% | 177 | 41.3% | 0.0003* |
| **29-38**  **(n= 135)** | No specific benefits in mind | 15 | 11.5% | 12 | 9.3% | 0.465 |
|  | Improved immunity | 42 | 32.3% | 36 | 27.9% | 0.306 |
|  | Improved general well-being | 87 | 66.9% | 75 | 58.1% | 0.536 |
|  | Improved mental health | 57 | 43.8% | 69 | 53.5% | 0.029* |
|  | Escape from daily routine | 16 | 12.3% | 37 | 28.7% | 0.001* |
|  | Staying fit, in shape | 15 | 65.4% | 74 | 57.4% | 0.126 |
|  | Pain alleviation | 51 | 11.5% | 15 | 11.6% | 1.054 |
|  | Stress reduction | 3 | 39.2% | 47 | 36.4% | 0.536 |
|  | Reduced lockdown fatigue | 8 | 2.3% | 39 | 30.2% | 0.0003* |
| **39-58**  **(n= 118)** | No specific benefits in mind | 8 | 6.7% | 17 | 15.2% | 0.041* |
|  | Improved immunity | 30 | 25.2% | 24 | 21.4% | 0.497 |
|  | Improved general well-being | 83 | 69.7% | 67 | 59.8% | 0.048 |
|  | Improved mental health | 34 | 28.6% | 39 | 34.8% | 0.137 |
|  | Escape from daily routine | 11 | 9.2% | 18 | 16.1% | 0.168 |
|  | Staying fit, in shape | 79 | 66.4% | 57 | 50.9% | 0.001* |
|  | Pain alleviation | 19 | 16% | 16 | 14.3% | 0.375 |
|  | Stress reduction | 29 | 24.4% | 29 | 25.9% | 0.669 |
|  | Reduced lockdown fatigue | 6 | 5% | 28 | 25% | 0.0001* |

*statistically significant data

**Physical Activity Before And During The COVID-19 Pandemic**

In connection with the research carried out, we kindly ask you to fill in the questionnaire below. The data obtained will be covered by professional secrecy and will be used for research purposes. We ask for participation of persons between 18 and 65 years of age.

Thank you for participating in the survey and we invite you to fill in the questionnaire.

Age: ………………
Gender: female male
Place of residence: city country
Level of education: elementary vocational secondary higher

1. How do you assess your well-being before and during the COVID 19 pandemic?

| **Assessment of well-being** | **Before the pandemic** | **During the pandemic** |
| --- | --- | --- |
| Very good |  |  |
| Good |  |  |
| Weak |  |  |
| Very weak |  |  |

2. How often did you undertake physical activity (PA) before and during the COVID 19 pandemic?

| **Frequency of PA** | **Before the pandemic** | **During the pandemic** |
| --- | --- | --- |
| Daily |  |  |
| Several times a week |  |  |
| Once a week |  |  |
| Several times a month |  |  |
| Less than once a month |  |  |
| None |  |  |

3.How long did your one-time physical activity last?

| **Duration** | **Before the pandemic** | **During the pandemic** |
| --- | --- | --- |
| Up to 10 minutes |  |  |
| 10-15 minutes |  |  |
| 15-30 minutes |  |  |
| 30-60 minutes |  |  |
| 60-90 minutes |  |  |
| Above 90 minutes |  |  |
| None |  |  |
|  | | |
|  | | |

4. What types of physical activity did you undertake before and during the pandemic? You can choose more than one answer.

| **Types of physical activity** | **Before the pandemic** | **During the pandemic** |
| --- | --- | --- |
| General improvement exercises |  |  |
| Marching, walks |  |  |
| Cycling/roller skating |  |  |
| Nordic walking |  |  |
| Strength exercises |  |  |
| Flexibility exercises/yoga |  |  |
| Fitness/pilates/aerobic |  |  |
| Jogging |  |  |
| None |  |  |

5.What health benefits did you expect to gain while taking up physical activity? You can choose more than one answer.

| **Types of benefits (motives)** | **Before the pandemic** | **During the pandemic** |
| --- | --- | --- |
| Improvement general well-being |  |  |
| Improved immunity |  |  |
| Improvement mental health |  |  |
| Reduced lockdown fatigue |  |  |
| Escape from daily routine |  |  |
| Pain alleviation |  |  |
| Stress reduction |  |  |
| Staying fit, in shape |  |  |
| No specific benefits in mind |  |  |
